# A Serological Biomarker of Versican Degradation is Associated with Mortality Following Acute Exacerbations of Idiopathic Interstitial Pneumonia

**DOI:** 10.1186/s12931-018-0779-y

**Published:** 2018-05-04

**Authors:** Jannie M. B. Sand, Yoshinori Tanino, Morten A. Karsdal, Takefumi Nikaido, Kenichi Misa, Yuki Sato, Ryuichi Togawa, Xintao Wang, Diana J. Leeming, Mitsuru Munakata

**Affiliations:** 1 Nordic Bioscience, Biomarkers and Research, Herlev Hovedgade, 205-207 Herlev, Denmark; 2 Department of Pulmonary Medicine, Fukushima Medical University, Fukushima, Japan

**Keywords:** Versican, Extracellular matrix, Biomarkers, Idiopathic interstitial pneumonia, Acute exacerbation

## Abstract

**Background:**

Idiopathic interstitial pneumonia (IIP) is characterized by an increased rate of extracellular matrix (ECM) remodeling resulting in fibrosis. Acute exacerbations of IIP represent periods of increased disease activity, thus we hypothesized that ECM remodeling was altered during acute exacerbations and investigated this by serological neo-epitope biomarkers.

**Methods:**

Patients who were sequentially admitted to the hospital with acute exacerbations of IIP were retrospectively analyzed for ECM remodeling at time of exacerbation (AE-IIP) and at clinical stability (S-IIP). Biomarkers released by matrix metalloproteinase-mediated degradation of collagen type I (C1M), III (C3M), IV (C4M), and VI (C6M), elastin (ELM7), versican (VCANM), biglycan (BGM), and C-reactive protein (CRPM) were assessed in serum by competitive ELISAs utilizing neo-epitope specific monoclonal antibodies.

**Results:**

Sixty-eight patients at AE-IIP and 29 at S-IIP were included in this retrospective analysis. Of these, 28 and 11 patients, respectively, had idiopathic pulmonary fibrosis. At AE-IIP, serum levels of C4M (*p* = 0.002) and C6M (*p* = 0.024) were increased as compared with S-IIP, while ELM7 (*p* = 0.024) and VCANM (*p* < 0.0001) were decreased. Lower VCANM levels at AE-IIP were associated with increased risk of mortality (HR 0.64 [95% CI 0.43–0.94], *p* = 0.022).

**Conclusions:**

The ECM remodeling profile was significantly altered during acute exacerbations of IIP, and a biomarker of versican degradation was related to mortality outcome. These results indicate that biomarkers of ECM remodeling may be useful in the non-invasive evaluation of acute exacerbations of IIP. Especially versican degradation, as measured serologically by VCANM, may have prognostic potential and help guide treatment for acute exacerbations.

## Background

Idiopathic interstitial pneumonia (IIP) is a subgroup of the diffuse parenchymal lung diseases (or interstitial lung diseases) characterized by inflammation and fibrosis of the alveolar structures [1]. This definition includes idiopathic pulmonary fibrosis (IPF), which has received special attention due to its severe nature and poor prognosis [2]. An acute exacerbation of IIP is an acute, clinically significant respiratory deterioration that is associated with disease progression and high risk of mortality [1, 3]. In IPF, up to 46% of deaths are preceded by an acute exacerbation and median survival time is as low as 3–4 months [3]. Acute exacerbations may accelerate the underlying fibrotic process, i.e. increase disease activity, and in that way contribute to disease progression. Acute exacerbations of IIP are hard to define and impossible to predict, thus diagnostic and prognostic biomarkers are needed.

Fibrosis, a hallmark of IIP, is characterized by extracellular matrix (ECM) deposition in the parenchyma (i.e. the interstitial matrix) and uncontrolled tissue remodeling. It results from an increased rate of ECM remodeling and a shift in the balance between protein synthesis and degradation, leading to ECM accumulation [4, 5]. ECM remodeling results in the release of protein fragments or neo-epitopes from the tissue. These may enter the circulation where levels can be assessed by specialized ELISAs as a measure of tissue turnover [6]. We have previously shown that serological levels of neo-epitope biomarkers are associated with the presence of an acute exacerbation of chronic obstructive pulmonary disease (COPD) [7, 8]. This indicates that rate of ECM remodeling is significantly altered during acute exacerbations of a chronic lung disease and that the group of serological neo-epitope biomarkers may be reflecting acute changes in disease activity. The association with disease activity is supported by the significant association of neo-epitope biomarkers with progression and mortality of IPF shown by Jenkins et al. in the PROFILE study [9].

Versican is a member of the chondroitin sulfate/dermatan sulfate proteoglycan family and is found in the ECM of soft tissues. It consists of a core protein with attached glycosaminoglycan (GAG) chains that can be sulfated to various degrees, with the pattern affecting cellular events and chemokine binding. Versican plays an important role in tissue homeostasis and is involved in tissue repair, ECM remodeling, inflammation, infection, and cell differentiation, migration, adhesion, and proliferation. Alterations in versican expression influences ECM compliance and the permeability of vessels and airways, and thus, it may be involved in various fibroproliferative disorders. The involvement of versican in chronic obstructive pulmonary disease, asthma, and bronchiolitis obliterans syndrome have been described (reviewed in [10]), underlining the importance of versican for upholding lung structure and function.

In this study, we wanted to investigate the changes in ECM remodeling during an acute exacerbation of IIP and hypothesized that the increased disease activity associated with acute exacerbations would lead to a change in the rate of ECM degradation. This was investigated by assessing ECM remodeling by measuring neo-epitopes in the circulation and evaluating their potential as biomarkers of acute exacerbations of IIP.

## Methods

### Study Design

This was a retrospective analysis of patients with IIP admitted to Fukushima Medical University Hospital between 2007 and 2014. All patients enrolled met the 2013 ATS/ERS Update of the International Multidisciplinary Classification of IIP [11], and were subsequently diagnosed as having IPF or IIP other than IPF (non-IPF). IPF was diagnosed using the definition in the 2011 ATS/ERS/JRS/ALAT joint statement [12] based on a pattern of usual interstitial pneumonia (UIP) on high resolution computed tomography (HRCT). Inclusion criteria for clinically stable IIP patients were no subjective symptoms of dyspnea or rapid deterioration on image findings for at least three months. Inclusion criteria for patients with acute exacerbation of IIP were progressive dyspnea within the last month; bilateral infiltrates or ground glass opacities on HRCT; decrease in PaO_2_ ≥ 10 Torr or PaO_2_/FiO_2_ < 300 mmHg [13]. Patients with pneumonia, heart failure, pulmonary thromboembolism, or pneumothorax were excluded, and so was patients whose progression was clearly associated with another disease. All patients included with an acute exacerbation underwent steroid pulse therapy.

The ethics committee at Fukushima Medical University approved this work (approval number 2484), and all clinical investigations were conducted according to the principles of the Declaration of Helsinki. Informed consent was not obtained as data were analyzed anonymously.

### Serological Biomarkers of ECM Remodeling

Blood samples were collected from patients upon diagnosis of IIP or at presentation with an acute exacerbation. All blood samples were immediately centrifuged and serum was aliquoted and stored at − 80 °C until biomarker analysis.

ECM remodeling was evaluated in serum by specific ELISAs (Nordic Bioscience, Herlev, Denmark) according to manufacturer’s instructions. Briefly, neo-epitope specific competitive ELISAs utilizing monoclonal antibodies were used to assess specific matrix metalloproteinase (MMP)-generated fragments of collagen type I (C1M [14]), III (C3M [15]), IV (C4M [16]), and VI (C6M [17]), elastin (ELM7 [18]), biglycan (BGM [19]), versican (VCANM [20]), and C-reactive protein (CRPM [21]). Reference biomarker levels were determined in a healthy population (*n* = 50) consisting of 48 (96%) females and 2 (4%) males with a mean age of 76 (SD 9).

### Statistical Analyses

Biomarker levels at stable disease and acute exacerbations were compared by Mann-Whitney test for independent samples or Wilcoxon test for paired samples. Spearman rank correlation was used to determine associations between biomarkers and clinical parameters. Univariate and multivariate Cox regression were used to determine the relationship between biomarker levels at time of acute exacerbation and mortality outcome for the total population and the IPF subpopulation. No single parameter was found significantly associated with mortality outcome in the IPF subpopulation, thus, biomarker analyses were performed uncorrected. Difference in survival time between biomarker tertiles at acute exacerbation was evaluated by Kaplan-Meier analysis. Maximal follow-up time was set to 100 days. This analysis was not performed in subpopulations due to the low number of patients. All data analysis were performed using MedCalc Statistical Software version 16.8.4 (MedCalc Software bvba, Ostend, Belgium). All tests were two-sided at the 0.05 level of significance and all *P* values are nominal as no adjustments were made for multiple comparisons.

## Results

### Basic Demographics

We included 29 patients with clinically stable IIP (S-IIP) and 68 patients with an acute exacerbation of IIP (AE-IIP). Of these, 11 and 28 patients, respectively, were diagnosed with IPF, and 28 patients had paired samples available at both clinically stable disease and at acute exacerbation. Basic demographics are summarized in Table 1. The acute exacerbation and stable groups were similar across the variables assessed with the exception of lung function measures which were significantly lower at acute exacerbation.

**Table 1:** Patient characteristics

	Total IIP population			IPF subpopulation			Paired IIP		
	S	AE	*p* value	S	AE	*p* value	S	AE	*p* value
n	29	68		11	28		28	28	
Age (mean SD)	69 (8)	71 (7)	0.206	67 (8)	71 (7)	0.150	68.3 (7.6)	68.3 (7.6)	NA
Female gender (n %)	6 (21%)	12 (18%)	0.726	0 (0%)	0 (0%)	NA	6 (21%)	6 (21%)	NA
Smokers (n %)									
Current	0 (0%)	1 (1%)	0.514	0 (0%)	0 (0%)	NA	0 (0%)	0 (0%)	NA
Ex	23 (79%)	50 (74%)	0.548	9 (82%)	23 (82%)	0.981	22 (79%)	22 (79%)	
Never	6 (21%)	17 (25%)	0.649	2 (18%)	5 (18%)	0.981	6 (21%)	6 (21%)	
FVC %pred (mean SD)	79 (27)	56 (19)	< 0.0001	73 (13)	55 (16)	0.0014	77 (26)	59 (24)	< 0.0001
VC (%pred), mean SD	78.7 (26.0)	54.6 (18.9)	< 0.0001	72.7 (13.5)	53.6 (16.0)	0.0023	76 (25)	57 (23)	< 0.0001
Time from IIP diagnosis to AE admission, months (median IQR)		12 (3–38)			29 (11–40)			24 (8–38)	
Treatment duration from AE admission, days (median IQR)		8 (3–14)			7 (2–12)			8 (4–16)	
Non-survivors at 100 days after AE admission (n %)		37 (54%)			13 (46%)			15 (54%)	
Time to death after AE admission, days (median IQR)		15 (6–35)			15 (4–43)			17 (10–30)	

Patient characteristics at time of stability (S) and acute exacerbation (AE) of idiopathic interstitial pneumonia (IIP) for the total population, and subpopulations of patients with idiopathic pulmonary fibrosis (IPF) or patients with paired samples (IPF *n* = 11). S and AE were compared for each population using Mann-Whitney test, Chi squared test, or Wilcoxon test as appropriate

### Biomarkers at Acute Exacerbation and Stability

At S-IIP, C3M, C4M, and VCANM serum levels were significantly correlated with age (Spearman's ρ of 0.431 [*p* = 0.0197], 0.401 [*p* = 0.0311], and − 0.557 [*p* = 0.0017], respectively). At AE-IIP, but not at S-IIP, serum C4M, C6M, and CRPM were significantly correlated with forced vital capacity (FVC) in percent of predicted value (0.373 [*p* = 0.0077], 0.358 [*p* = 0.0108], and 0.329 [*p* = 0.0197], respectively). Serum levels of VCANM at AE-IIP correlated significantly with survival time (0.265 [*p* = 0.0292]).

**Fig. 1:**
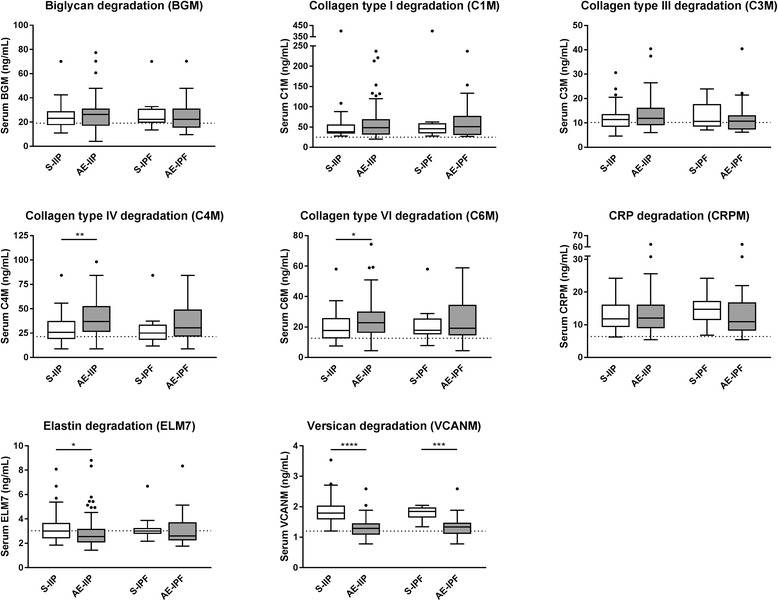
Serum biomarker levels at stability and acute exacerbations. Biomarker levels are shown in the total IIP population (left) and the IPF subpopulation (right) at stability (S, white) and at acute exacerbation (AE, grey). Data are shown as Tukey box-plots where the box represents the interquartile range (IQR) with the median shown as a line, whiskers represent 1.5 times the IQR, and circles show data points outside this range. Dotted line indicate reference biomarker level of a healthy population. Statistical significance was determined by Mann-Whitney test (*p < 0.05, **p < 0.01, ***p < 0.001, ****p < 0.0001)

**Fig. 2:**
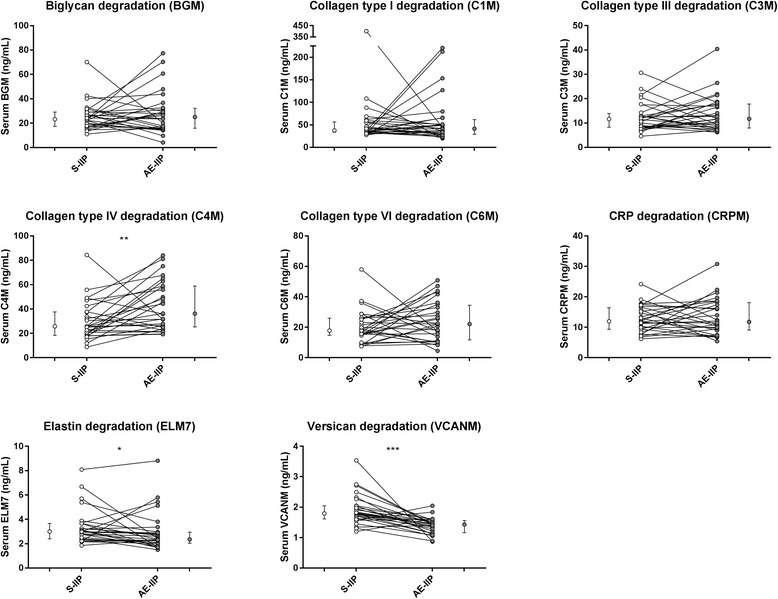
Paired serum biomarker data for the IIP population at acute exacerbation and stability. Data are shown as line plots at stability (S-IIP, white) and at acute exacerbation (AE-IIP, grey) for the paired IIP population (*n* = 28), with adjacent median value with interquartile range (IQR). Statistical significance was determined by paired Wilcoxon test (*p < 0.05, **p < 0.01, ***p < 0.001)

We then compared serum levels of biomarkers of ECM remodeling at stable disease and acute exacerbations. In the total population of IIP, we found that serum levels of C4M (median 36.9 [IQR 26.4–52.0] vs. 25.8 [19.1–37.4] ng/mL, *p* = 0.0019) and C6M (22.9 [16.6–30.0] vs. 17.8 [13.5–25.7] ng/mL, *p* = 0.0235) were significantly elevated at AE-IIP as compared with S-IIP (Fig. 1). Conversely, serum levels of ELM7 (2.55 [2.07–3.18] vs. 3.01 [2.42–3.67] ng/mL, p = 0.0235) and VCANM (1.29 [1.08–1.45] vs. 1.79 [1.59–2.04] ng/mL, *p* < 0.0001) were significantly decreased at AE-IIP. In the IPF subpopulation, only VCANM levels (1.34 [1.12–1.47] vs. 1.84 [1.66–1.97] ng/mL, *p* = 0.0001) were significantly decreased at AE-IIP as compared with S-IIP (Fig. 1). In the paired population, C4M levels were significantly elevated at AE-IIP (36.2 [25.6–58.7] vs. 25.8 [18.8–37.5] ng/mL, *p* = 0.0036) while ELM7 (2.35 [2.05–2.93] vs. 2.99 [2.41–3.66] ng/mL, *p* = 0.0362), and VCANM (1.43 [1.18–1.55] vs. 1.79 [1.62–2.04] ng/mL, *p* = 0.0001) were significantly decreased as compared with levels at S-IIP (Fig. 2).

### Prediction of Mortality Following an Acute Exacerbation

**Fig. 3:**
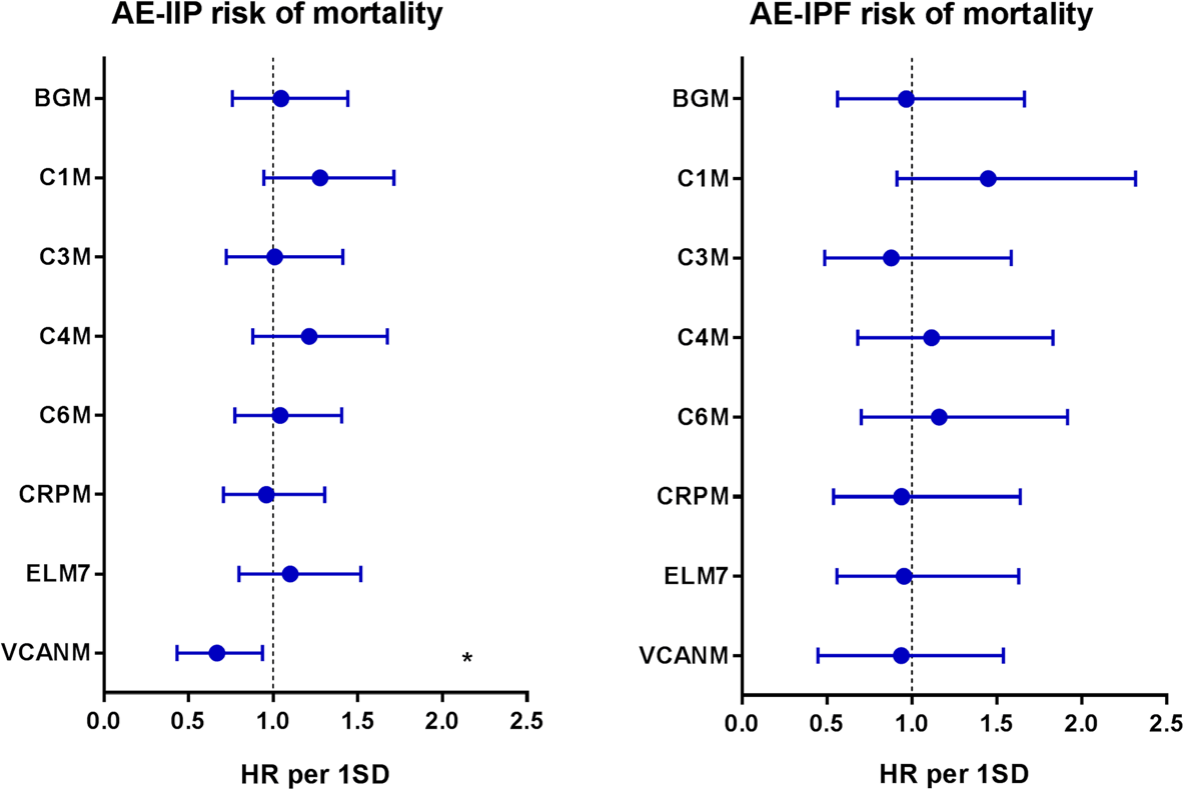
Biomarkers as predictors of mortality following acute exacerbations of IIP or IPF. Cox regression analysis for mortality risk presented as hazard ratio (HR) with 95% confidence intervals for one standard deviation (SD) increase in biomarker levels at time of acute exacerbation for the total population of IIP (left) and the subpopulation of IPF (right). HR for IIP was corrected for age and gender. Asterisks indicate statistical significance (*p < 0.05)

**Fig. 4:**
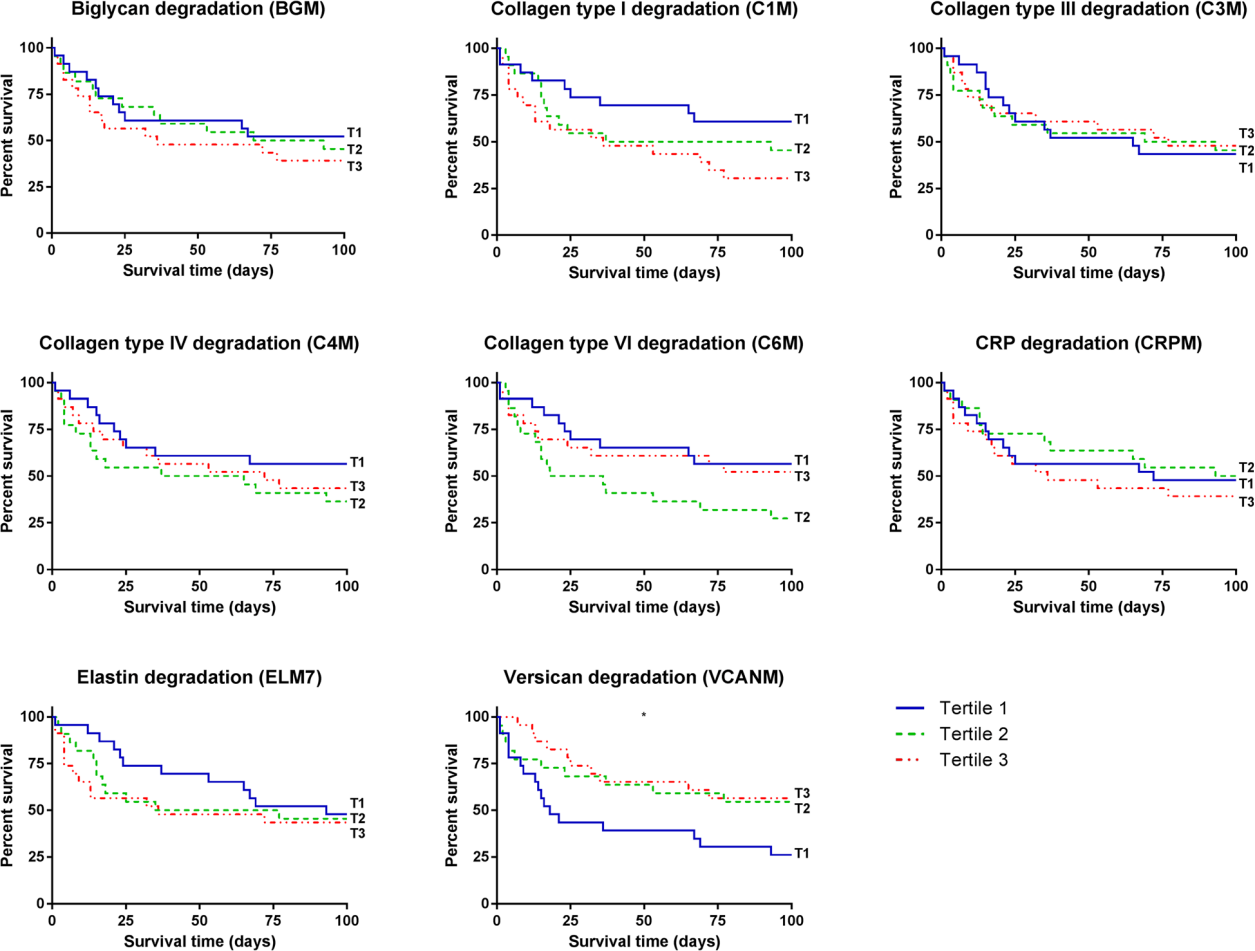
Kaplan-Meier curves for survival by biomarker tertiles at time of acute exacerbation of IIP. Asterisks indicate statistical significance between the lowest (T1), middle (T2), and highest (T3) biomarker tertiles (*p < 0.05)

Univariate Cox regression was used to determine the clinical parameters that were related to mortality following an acute exacerbation of IIP (Table 2). Older age and female gender were significantly associated with a higher risk of mortality in IIP, but not in the IPF subpopulation, with hazard ratios of 1.059 (95% CI 1.007-1.113; *p* = 0.0248) and 2.420 (95% CI 1.166-5.025; *p* = 0.0177), respectively. These two significant variables were included as covariates in multiple Cox regression analysis of the relationship between biomarker levels at AE-IIP and mortality outcome (Fig. 3 and Table 2). Here we found that lower levels of VCANM at AE-IIP was associated with a higher risk of mortality with a hazard ratio of 0.636 (95% CI 0.432-0.937) per SD increase in serum VCANM levels (*p* = 0.0219). No biomarkers were significantly associated with mortality risk in the IPF subpopulation (Fig. 3 and Table 2). Survival times for IIP patients belonging to the different tertiles of biomarkers at AE-IIP were compared by Kaplan-Meier curves (Fig. 4). The lowest tertile of VCANM was associated with significantly shorter survival time as compared to the two higher tertiles (*p* = 0.0397).

**Table 2:** Clinical variables and biomarkers as predictors of mortality following acute exacerbations of IIP

Variable	AE-IIP total population (*n* = 68)		AE-IPF subpopulation (*n* = 28)	
	HR (95% CI)	*p* value	HR (95% CI)	*p* value
Clinical variables				
Age	1.059 (1.007–1.113)	0.025*	1.045 (0.952–1.147)	0.357
FVC %pred	0.985 (0.961–1.008)	0.195	1.023 (0.991–1.057)	0.164
Time from diagnosis to admission, months (median IQR)	1.010 (0.999–1.020)	0.066	1.013 (0.991–1.034)	0.255
Treatment duration from admission, days (median IQR)	0.961 (0.915–1.009)	0.110	0.940 (0.856–1.031)	0.187
Female gender	2.420 (1.166–5.025)	0.018*	NA	NA
Ex smoker	0.631 (0.312–1.279)	0.202	1.120 (0.248–5.054)	0.883
Current smoker	0.000 (5× e^− 191^ - 963× e^177^)	0.957	NA	NA
Biomarkers				
BGM	1.047 (0.759–1.442)	0.783	0.966 (0.562–1.662)	0.901
C1M	1.277 (0.946–1.714)	0.108	1.449 (0.911–2.316)	0.116
C3M	1.010 (0.722–1.412)	0.955	0.878 (0.486–1.585)	0.665
C4M	1.214 (0.879–1.675)	0.237	1.115 (0.681–1.831)	0.663
C6M	1.042 (0.774–1.406)	0.785	1.160 (0.702–1.915)	0.564
CRPM	0.959 (0.707–1.304)	0.792	0.939 (0.538–1.638)	0.824
ELM7	1.101 (0.798–1.518)	0.559	0.954 (0.559–1.628)	0.862
VCANM	0.636 (0.432–0.937)	0.022*	0.828 (0.446–1.538)	0.551

The predictive value of clinical variables for mortality following an acute exacerbation of idiopathic interstitial pneumonia (IIP, *n* = 68) or the subpopulation of idiopathic pulmonary fibrosis (IPF, *n* = 28) was evaluated by univariate Cox regression. The predictive value of biomarkers measured at time of acute exacerbation for mortality outcome was evaluated by multiple Cox regression adjusting for age and gender for IIP and by univariate Cox regression for IPF. Data are shown as mean hazard ratio (HR) with 95% confidence intervals (CI). HR for biomarkers are shown per one standard deviation (SD) increase in biomarker levels at time of acute exacerbation. Asterisks indicate statistical significance (*p < 0.05)

## Discussion

Here we have demonstrated for the first time that acute exacerbations of IIP are associated with acute changes in remodeling of the lung ECM and that these are related to outcome. Our main findings were: degradation of collagen type IV (C4M) and VI (C6M) was significantly elevated during an acute exacerbation of IIP as compared with stable disease, while degradation of elastin (ELM7) and versican (VCANM) was significantly decreased; and lower levels of versican degradation (VCANM) during an AE-IIP was significantly associated with increased risk of mortality.

Increased degree of epithelial injury during AE-IIP has previously been suggested [22] and is supported by the finding of elevated KL-6 and SP-D plasma levels in AE-IPF as compared with stable IPF [23]. In the current study, we found increased levels of C4M and C6M, indicating an increased rate of degradation of collagen type IV and VI during acute exacerbations. Collagen type IV is the main component of the basement membrane, located beneath the epithelial layer and collagen type VI is closely associated with the basement membrane, linking it to the interstitial matrix. Injury to the protective epithelial layer is likely to disrupt the underlying basement membrane, resulting in release of degradation fragments from collagen type IV and VI. Thus, our data support the hypothesis that epithelial injury is increased during acute exacerbations of IIP.

Serum levels of VCANM were significantly decreased during AE-IIP as compared to the stable state, indicating a potential build-up of versican in the interstitial lung tissue during an acute exacerbation. Previous results showed decreased serum levels of another proteoglycan, syndecan-4, during AE-IIP [24]. To assess syndecan-4 in serum, the transmembrane protein needs to be shed from the cell surface by MMPs and move to the bloodstream. For this to happen, the protein must undergo proteolytic processing, which could indicate that the degradation of both syndecan-4 and versican is decreased during an acute exacerbation of IIP. Increased versican deposition has been demonstrated in fibroblastic foci of IPF patients [25, 26] as well as in the lungs of patients with COPD [27, 28] or asthma [29], suggesting a role in the faulty repair processes related to chronic lung diseases. Serum VCANM levels at AE-IIP are comparable to the reference level seen in a healthy, unmatched population. One could speculate that an increased rate of degradation during stable disease is an attempt to normalize the increased versican levels found in the lungs. This response might be halted temporarily while the body fights off the acute effects of an exacerbation, resulting in normal levels of versican turnover. Versican has been demonstrated to inhibit the function of elastin-binding protein, resulting in impaired formation of elastic fibers [27, 30]. Thus, the accumulation of versican may have a negative impact on lung and vessel structure. The poorer quality of elastic fibers may lead to a decreased degradation of elastin, as indicated by lower levels of ELM7, as an attempt to prevent further loss of functioning elastic fibers.

Versican is involved in the inflammatory response, and the macrophage-derived versican expression has been found increased in response to acute inflammation [31]. IIP is a chronic condition that may show altered protein expression, whereas an acute exacerbation is an acute state of inflammation leading to a different protein expression pattern. A mouse model of acute lung injury induced by polyinosine-polycytidylic acid to mimic a viral infection has shown accumulation of versican in the lung. Furthermore, versican knock-out mice had an attenuated proinflammatory response [32]. These data could indicate that versican may build up in the lungs during acute exacerbations of chronic lung diseases, which is in line with a decrease in its degradation seen in the present study. Additionally, a decrease in versican degradation has also been associated with acute exacerbations of COPD [7].

The major limitations to this study are related to the retrospective nature of the study design and the heterogeneous study population. The diagnosis of IIP includes different fibrotic lung diseases, which may introduce a greater variation in the present data. We have diagnosed patients with an UIP pattern on HRCT as having IPF using the definition in the 2011/ATS/ERS/JRS/ALAT joint statement. Because surgical lung biopsy was not performed, a histopathological analysis to confirm the UIP pattern was not possible, and the non-IPF patients in the current study represents a heterogeneous population. It has been debated whether a possible UIP pattern on HRCT is sufficient to make a diagnosis of IPF [33–35], and one study found 23% of patients with HRCT findings inconsistent with the UIP pattern to have a definite/probable UIP pattern on biopsy. Although idiopathic non-specific interstitial pneumonia (NSIP) might be the major type of IIP in the non-IPF group in the present study, it is possible that other IIPs such as IPF and unclassifiable IIPs were included. As a definite diagnosis could not be made in the non-IPF patients, subgroup analysis was only performed for the IPF group. Future studies should include more patients and perform subgroup analysis on IPF and NSIP to evaluate if ECM remodeling differs between these patient groups. Another limitation to this study is the inclusions of patients with unpaired samples or paired samples taken far apart. We have compared baseline characteristics for all patients to investigate the effect of inter-patient variation in the total population, IPF only as well as for patients with paired samples. Only lung function differed significantly between stable disease and time of exacerbations, indicating that the included patients were comparable as lung function is expected to be affected by an acute exacerbation. However, it cannot be ruled out that a lower lung function during acute exacerbation may be related to a permanent change and thus disease progression. We compared unpaired samples and subsequently confirmed the findings for C4M, ELM7 and VCANM using paired samples. Only the association with C6M could not be confirmed using paired samples. For patients with paired samples, the follow-up time between stable disease and acute exacerbation was long which may have influenced the disease progression and activity.

## Conclusion

In conclusion, our data indicate that acute exacerbations of IIP are associated with degradation of the basement membrane and a build-up of versican and elastin in the interstitial matrix of the lung, as determined by serological neo-epitope specific biomarkers. The difference in degradation profile for the proteins studied is intriguing and indicate activation of different processes contributing to AE-IIP. Furthermore, the potential build-up of versican indicated by lower levels of VCANM, a biomarker reflecting versican degradation, is associated with increased risk of mortality. Thus, serological neo-epitope biomarkers reflecting lung ECM remodeling may be useful tools in the non-invasive assessment of acute exacerbations of IIP, and VCANM may represent a novel prognostic biomarker. However, the current data should be validated in separate cohorts to evaluate the potential use.

## Abbreviations

AE-IIP: Acute exacerbation of idiopathic interstitial pneumonia
BGM: Biglycan degraded by MMPs
C1M: Collagen type I degraded by MMPs
C3M: Collagen type III degraded by MMPs
C4M: Collagen type IV degraded by MMPs
C6M: Collagen type VI degraded by MMPs
COPD: Chronic obstructive pulmonary disease
CRPM: C-reactive protein degraded by MMPs
ECM: Extracellular matrix
ELM7: Elastin degraded by MMP7
FVC: Forced vital capacity
GAG: Glycosaminoglycan
HRCT: High resolution computed tomography
IIP: Idiopathic interstitial pneumonia
IPF: Idiopathic pulmonary fibrosis
IQR: Interquartile range
MMP: Matrix metalloproteinase
NSIP: Non-specific interstitial pneumonia
S-IIP: Clinically stable idiopathic interstitial pneumonia
VCANM: Versican degraded by MMPs
